# A New Strategy for Effective Succinic Acid Production by *Enterobacter* sp. LU1 Using a Medium Based on Crude Glycerol and Whey Permeate

**DOI:** 10.3390/molecules24244543

**Published:** 2019-12-12

**Authors:** Marcin Podleśny, Jakub Wyrostek, Jagoda Kucharska, Piotr Jarocki, Elwira Komoń-Janczara, Zdzisław Targoński

**Affiliations:** 1 Department of Biotechnology, Microbiology and Human Nutrition, University of Life Sciences in Lublin, 8 Skromna Street, Lublin 20-704, Poland; podlesnymarcin@hotmail.com (M.P.);; 2 Department of Analysis and Food Quality Assessment, University of Life Sciences in Lublin, 8 Skromna Street, Lublin 20-704, Poland

**Keywords:** succinic acid, *Enterobacter*, crude glycerol, whey permeate

## Abstract

The newly-isolated strain *Enterobacter* sp. LU1, which has previously been shown to be an effective producer of succinic acid on glycerol with the addition of lactose, was used for further intensive works aimed at improving the production parameters of the said process. The introduction of an initial stage of gentle culture aeration allowed almost 47 g/L of succinic acid to be obtained after 168 h of incubation, which is almost two times faster than the time previously taken to obtain this amount. Furthermore, the replacement of glycerol with crude glycerin and the replacement of lactose with whey permeate allowed the final concentration of succinic acid to be increased to 54 g/L. Considering the high content of yeast extract (YE) in the culture medium, tests were also performed with a reduced YE content via its partial substitution with urea. Although this substitution led to a deterioration of the kinetic parameters of the production process, using the fed-batch strategy, it allowed a succinic acid concentration of 69 g/L to be obtained in the culture medium, the highest concentration ever achieved using this process. Furthermore, the use of microaerophilic conditions meant that the addition of lactose to the medium was not required, with 37 g/L of succinic acid being produced on crude glycerol alone.

## 1. Introduction

The success of any biotechnological project is largely determined by the presence of an efficient biocatalyst that is capable of utilizing cheap and renewable substrates at the catalyst’s optimal process conditions. Hence, the cost-effectiveness of a biotechnological process is inseparably linked to the use of cheap raw materials. After cost-effectiveness, the most important factors for a basal substrate in a biotechnological project are that it be renewable and that it has the lowest possible competitiveness with food production. Recently, there has been increasing interest regarding the application of glycerol (a tri-hydroxyl alcohol) as a substrate in biotechnological production (e.g., of succinic acid). In nature, glycerol occurs mainly in the form of esters with fatty acids, and is the main component of animal lipids and vegetable oils [1]. The production of glycerol requires the hydrolysis of ester bonds in triglycerides, which results in the synthesis of free fatty acids that may be further used for the production of soap or biodiesel via saponification and transesterification, respectively [2]. As well as its relatively low price, glycerol has various other advantages for use in biotechnological processes. For example, glycerol’s high degree of carbon atom reduction (κ = 4.67, where κ denotes the degree of reduction to one atom of carbon and represents the number of available electrons per atom of carbon in a molecule) is a highly valuable trait in the production of certain chemicals (e.g., ethanol or succinic acid; for comparison, the carbon atom reduction of glucose and xylose is 4). Additionally, the use of glycerol as a source of carbon in succinic acid production reduces by-product formation [3]. Glycerol can also be applied in many other biotechnological processes such as the production of 1,3-propanediol by *Clostridium butyricum* [4], the production of propionic acid by *Propionibacterium acidipropionici* [5], the production of 2-3-butanediol by *Enterobacter cloacae* [6], or the production of ethanol by *Escherichia coli* [7]. Despite the aforementioned advantages of glycerol as a substrate for the biotechnological production of organic low-molecular-weight compounds, its utilization by most microorganisms as the sole source of carbon under anaerobic conditions is significantly limited when there is no access to electron acceptors other than oxygen (e.g., nitrates or fumaric acid). However, in most cases, the application of these electron acceptors reduces the production of succinic acid at the expense of the overproduction of, for example, acetic acid (use of nitrates, aerobic process), or else is unsuitable due to economic concerns (fumaric acid production). For instance, the final concentration of succinic acid produced on glycerol (i.e., with no additional electron acceptors) using the bacterium *Anaerobiospirillum succiniciproducens* in a periodic culture was ca. 4.9 g/L culture medium [3]. For comparison, the same microorganism cultured on glucose achieved a succinic acid production of ca. 33 g/L [8]. A similar situation is observed with *Actinobacillus succinogenes;* its culturing on glucose allows the production of ca. 66 g/L of succinic acid, whereas its culturing on glycerol allows a production of ca. 10 g/L [9]. A recent study reported a colossal improvement of glycerol consumption by bacteria of the genus *Enterobacter* under anaerobic conditions through the addition of lactose [10]. Interestingly, the main product of this co-fermentation was succinic acid. The above-described observations indicate the potential for the intensification of succinic acid production using bacteria of the genus *Enterobacter*. 

Multiple strategies have been applied in the biotechnological production of succinic acid. Many involve periodic batch cultures without the addition of nutrients during culturing and with the removal of metabolites. Considering the necessity of maintaining various culture parameters (e.g., pH, temperature, stirring rate, CO_2_ access) at a stable level in order to achieve optimal and repeatable results, these cultures are usually run in bioreactors of different sizes [11,12,13]. An equally common method of succinic acid production involves periodic fed-batch cultures, also known as semi-periodic cultures. In such cases, lacking nutrients are added during the course of culturing. This culturing method is indispensable when, for example, a substrate used in high concentrations has an inhibiting effect on the cultured microorganism and the productivity of the process (the ratio of the amount of product formed to the amount of substrate consumed) does not allow a desirable concentration of succinic acid to be produced from the initially added substrate [14,15,16]. A new approach involves the co-production of succinic acid and ethanol by simultaneous saccharification and co-fermentation [17]. However, in this process, alcohol accounted for the large majority of the product, 120 g/L, while the succinic acid concentration was 34.84 g/L.

In the bacterial production of succinic acid, another significant factor is the method that is used to neutralize the acid that is produced. The most frequently applied neutralizing agents include sodium carbonate, sodium bicarbonate, magnesium carbonate, and ammonia water. Additionally, considering the large amounts of base that are added during fermentation, a further significant factor is the type of metal ion that constitutes the base as this can affect the consumption of substrates during the process and the quantities of the final products [18]. For instance, sodium ions play a role in maintaining the transmembrane pH gradient and in regulating intracellular pH [19]. Excessive quantities of calcium ions have a negative effect on the growth kinetics of bacterial biomass by disturbing the transport of substrates and metabolites through the cell membrane [20]. The presence of magnesium ions has a positive impact on the metabolic activity of microorganisms during succinic acid production, as they are co-factors of the phosphoenolpyruvate carboxykinase enzyme, which is highly significant for this process [21]. Additionally, the use of NH_4_^+^ ions in the neutralization process reduces the growth of bacterial biomass and inhibits the production of succinic acid, despite the application (at the purification stage) of a process that leads to the production of ammonium sulfate (which may be sold as fertilizer). For example, it has been observed that, in the case of *E. coli* bacteria, a concentration of ammonium ions above 10 g/L caused a drastic decrease in both bacterial biomass growth and succinic acid synthesis [22]. Consequently, to achieve the highest production rates in the microbial production of succinic acid, compounds containing sodium ions [23] or magnesium ions [11,24] are most often applied. In this research work, we present the intensification of the production of succinic acid using a novel isolate *Enterobacter* sp. LU1 [10]. The fermentation was conducted with the use of crude glycerol obtained from biorefinery producing biodiesel and whey permeate containing lactose produced in a dairy plant. We hope that the results presented in this work are promising for future implementation of the new succinic acid fermentation strategy in industrial practice.

## 2. Materials and Methods

### 2.1. Materials

Crude glycerol was obtained from LOTOS Biofuels (Czechowice, Poland) and contained approx. 85% of glycerol, 2.6–3.5% of inorganic salts (including NaCl), 3–6% of MONG (matter organic non glycerol comprised of fatty acid methyl esters, fatty acid ethyl esters, free fatty acids and glycerides), <1% of elements (e.g., K, Ca, Mg) and water. The composition of the specific portions of crude glycerol were determined in the biorefinery. Powdered whey permeate was obtained from the Ostrowia Production Plant (Ostrowia, Poland) and contained about 77.0% lactose. All other chemicals (including glycerol 99.6%) were of analytical grade and commercially available.

### 2.2. Microorganism and Culture Conditions

*Enterobacter* sp. strain LU1 was kept at −80 °C with 20% (*w*/*w*) glycerol added. Inoculum cultures were grown anaerobically for 6 h in 100 mL serum bottles with gas-tight butyl rubber stoppers at 37 °C in brain heart infusion medium (BHI) (OxoidThermo Scientific, Basingstoke, UK) at a pH of 7.4. For the fermentations with a decreased amount of yeast extract (YE) that were conducted under microaerobic conditions, overnight semi-aerobic cultures of *Enterobacter* sp. LU1 on minimal medium were used as a direct inoculum. The minimal medium had a pH of 7.6 and contained crude glycerol (30 g/L) as the sole carbon source without YE, and the other constituents were as follows (g/L): urea, 2.0; MgSO_4_ × 7H_2_O, 0.2; MgCO_3_, 5.0; CaCl_2_, 0.5; K_2_HPO_4_, 1.0; and NaCl, 1.0.

### 2.3. Batch and Fed-Batch Fermentations

The pre-culture of *Enterobacter* sp. LU1 (200 mL) was inoculated in a Biostat A fermenter (Sartorius AG, Goettingen, Germany) containing 1.8 L of fermentation medium. Some of the components of the culture medium differed in concentration between the experiments described below. This applies to CaCl_2_ and MgSO_4_ and results from simultaneous studies optimizing the nutrient composition. The temperature of fermentation was set at 34 °C and stirring was performed at 250–500 rpm. To avoid the pH dropping below 7.0, the pH was adjusted automatically with solutions of 5% NaOH (*w*/*v*) and 20% Na_2_CO_3_ (*w*/*v*) or was adjusted manually by the gradual addition of sterile magnesium carbonate. In the aerobic stages of fermentation and when microaerobic conditions were used, the volume of air delivered to bioreactor was about 1 vvm. 

### 2.4. Description of Experiments

#### 2.4.1. Increase of Lactose Content in Fermentation Medium

In the first stage of the study, the lactose content in the fermentation medium was 25.0 g/L while the glycerol analytical grade concentration was maintained at 50 g/L. The remaining components of the culture medium were as in the culture used in [10], namely (g/L): YE, 15.0; MgCO_3_, 5.0; Na_2_HPO_4_, 0.31; NaH_2_PO_4_ × H_2_O, 1.16; MgCl_2_ × 6H_2_O, 0.2; CaCl_2_, 0.15; and NaCl, 1.0. In a subsequent experiment, the lactose content in the culture medium was increased to 32.5 g/L, while the glycerol concentration remained at a predetermined level of 50 g/L. The cultivation conditions in the bioreactor remained unchanged.

#### 2.4.2. Fermentation Medium Based on Crude Glycerol and Whey Permeate

Additionally, an experiment was conducted that involved the use of crude glycerol and lactose originating from whey permeate. Both substrates were added to the prepared microbiological medium in doses that resulted in their concentrations in the culture medium being 50 g/L for glycerol and 32.5 g/L for lactose. The fermentation medium was adjusted to avoid its pH dropping below 7.0 by the periodic addition of sterile powdered MgCO_3_. The initial concentration of MgCO_3_ in the culture medium was 30 g/L. Due to the presence of various micro- and macroelements in the crude glycerin and whey permeate, only CaCl_2_ was added to the culture medium at a dose of 0.15 g/L. The nitrogen source was YE at a concentration of 15 g/L

#### 2.4.3. Reduced Yeast Extract Content and Flask Based Inoculum

In order to investigate the effect of reducing the YE concentration in the fermentation medium, two fermentations were performed. For the first fermentation, inoculum was prepared under aerobic conditions on minimal culture medium with crude glycerol used as the sole source of carbon (30 g/L). The bioreactor was inoculated with 100 mL of an overnight culture. Before the addition of the inoculum, the following were introduced into the bioreactor: (1) 1.9 L of a fermentation medium with the following composition (g/L): crude glycerol, 58.8; whey permeate, 43.5; urea, 1.0; NaCl, 0.05; CaCl_2_, 0.05; K_2_HPO_4_, 1.0; MgSO_4_ × 7H_2_O, 0.1; MgCO_3_, 5.0; and (2) an anti-foaming agent contained in a dose of 2 mL/L of the same fermentation medium. After four hours of aeration, the stirrer speed was decreased to 250 rpm, system aeration was ceased, and the following compounds were added (g/L): urea, 2.0; YE, 1.0; K_2_HPO_4_, 1.0; and MgCO_3_, 20.0. Before the end of the second day of culturing, the culture was supplemented with 1 g/L YE, 0.63 g/L K_2_HPO_4_ and 0.5 g/L urea, and the pH was increased through the addition of 10 g/L MgCO_3_. At the beginning of the eighth day (170 h), YE, K_2_HPO_4_, and CO(NH_2_)_2_ were added to the culture at a dose of 1 g/L each. On the next day of culturing (193 h), 14.7 g/L of whey permeate and 10 g/L of MgCO_3_ were introduced into the bioreactor (bringing the total concentration of magnesium carbonate in the bioreactor to 45 g/L).

#### 2.4.4. Reduced YE Content and Bioreactor Based Inoculum

Another bioreactor culture was prepared with a decreased YE concentration, with which the production of succinic acid was begun via inoculum proliferation in the bioreactor. The pre-inoculum included 200 mL of the aerobic culture based on 30 g/L of crude glycerol. The multiplied *Enterobacter* sp. LU1 strain bacteria were transferred to the bioreactor together with the post-culture liquid. The culture was aerated at 1 vvm in order to provide the best possible conditions for the proliferation of a high volume of biomass, and the stirrer speed was set at 400 rpm. The composition of the proliferating medium in the bioreactor was as follows (g/L): crude glycerol, 58.8; NaCl, 1.0; K_2_HPO_4_, 1.0; MgSO_4_ × 7H_2_O, 0.32; CaCl_2_, 0.5; urea, 1.5; and MgCO_3_, 5.0. On the next day of culturing, after 24 h of aeration, the aerobic stage was completed. Then, 200 mL of the culture was left in the bioreactor and a new culture medium was added and the stirrer speed was reduced to 250 rpm. The pH was adjusted only with the addition of magnesium carbonate. The composition of the fermentation medium was as follows (g/L): crude glycerol, 58.8; whey permeate, 43.5; urea, 1.0; YE, 2.0; K_2_HPO_4_, 1.0; and MgCO_3_, 30.0. After 42 h of anaerobic culturing, 1 g/L of urea and 1 g/L of K_2_HPO_4_ were added to the bioreactor. After 90 h (on the fifth day of culturing), the bioreactor was fed with a new dose of urea (1 g/L) and K_2_HPO_4_ (1 g/L) as well as with MgCO_3_ (20 g/L). At the beginning of the sixth day (121 h), whey permeate (ca. 15 g/L) was added to the bioreactor due to the fact that lactose was consumed faster than glycerol. On the seventh day, urea (1 g/L), dipotassium hydrogen phosphate (1 g/L), and magnesium carbonate (20 g/L) were added again. In the 189th hour, 30 g/L of whey permeate and 30 g/L of crude glycerol were added, followed by the addition of 10 g/L of magnesium carbonate (bringing the total amount of MgCO_3_ added in the anaerobic stage to 80 g/L), 1 g/L of urea, and 1 g/L of K_2_HPO_4_. The culture was terminated before the end of the 12th day. 

#### 2.4.5. Microaerobic Cultivation on Crude Glycerol and Whey Permeate

For the microaerobic cultivations, the first of the substrates (glycerol) was added in the form of crude glycerol, whereas the other (lactose) was contained in whey permeate. As in the previous cultures, the inoculum included 200 mL of the flask culture of the test strain conducted under aerobic conditions on the minimal medium with crude glycerol applied as the source of carbon (30 g/L). The overnight cultures were transferred into a prepared bioreactor that contained 1.8 L of culture medium (crude glycerol, 60 g/L; whey permeate, 60 g/L; K_2_HPO_4_, 1.0 g/L; MgSO_4_ × 7H_2_O, 0.32 g/L; urea, 1.5 g/L; and MgCO_3_, 10 g/L). After two days, the stirring rate was increased from 300 rpm to 400 rpm. After three days, the pH value dropped to 6.6, and it was therefore adjusted to 7.5 using ammonium water. At the same time, 10 g/L of magnesium carbonate was added.

#### 2.4.6. Microaerobic Cultivation with Glycerol as the Sole Carbon Source

In another experiment, which was run in semi-aerobic conditions, technical glycerol (crude) was used as the only source of carbon. Inoculum was prepared as in the previous cultures. The fermentation medium contained (g/L): crude glycerol, 60; K_2_HPO_4_, 1.0; MgSO_4_ × 7H_2_O, 0.32; urea, 1.5; CaCl_2_, 0.5; and MgCO_3_, 10. From the beginning, the bioreactor culture was run under microaerophilic conditions (aeration at 0.25 vvm) with stirring at 300 rpm. The culture liquid was neutralized with magnesium carbonate fed in doses of 10 g/L. At the end of the second day, an additional portion of glycerol and inorganic salts was added (crude glycerol, 60 g/L; K_2_HPO_4_, 1.0 g/L; MgSO_4_ × 7H_2_O, 0.32 g/L; CaCl_2_, 0.5 g/L; and MgCO_3_, 10 g/L). Glycerol analytical grade was used as a control to investigate the potential toxicity of the crude glycerol impurities. After four days, the stirring rate was increased to 400 rpm. On the fifth day of culturing, an additional portion 1.0 g/L of dipotassium hydrogen phosphate was fed into the system. After seven days of culturing, the total amount of MgCO_3_ fed into the bioreactor had reached 55 g/L.

### 2.5. Analytical Methods

Cell growth was monitored by measuring the absorbance of the broth at 600 nm (OD600) after diluting the sample 1:1 with 7% HCl (*v*/*v*). The biomass concentration of the *Enterobacter* sp. LU1 strain was estimated by determining the cell dry weight (CDW) using a predetermined correlation curve obtained between the absorbance measured at 600 nm and the cell dry weight (g/L). Samples for the detection of succinic acid and by-products were prepared by centrifugation of the culture broth at 6000 g for 5 min. After dilution with water (1:1), the resulting supernatant was analyzed by a high-performance liquid chromatography system with an Agilent HP 1100 DAD detector G1315A and autosampler model 234 (Gilson, Middleton, WI, USA) equipped with an Aminex HPX-87H ion-exchange column (Bio-Rad, Hercules, CA, USA) and a refractive index detector using 0.03 M sulfuric acid as the mobile phase at 42 °C [25].

### 2.6. Calculation of Fermentation Parameters

The amounts of succinate and by-products in the culture broth were used for calculations of the mass yield of succinic acid (Y_SA_), and the volumetric succinate productivity (Q_SA_). Mass yield of succinate (Y_SA_), expressed in grams per gram from glycerol and lactose or glycerol alone (microaerobic cultivations), was calculated from

Y_SA_ = P/S (1)

The volumetric succinate productivity (Q_SA_), expressed in grams per liter per hour, was calculated from

Q_SA_ = P/V × *t* (2)

where P is the amount of succinate in the culture liquid at the end of a cultivation (in grams); S is the total amount of carbon sources (glycerol and lactose or glycerol alone) consumed (in grams); V is the initial volume of culture liquid (in liters); and *t* is the fermentation duration (in hours).

## 3. Results

### 3.1. Increase of Lactose Content in Fermentation Medium and Usage of Crude Glycerol and Whey Permeate

**Figure 1:**
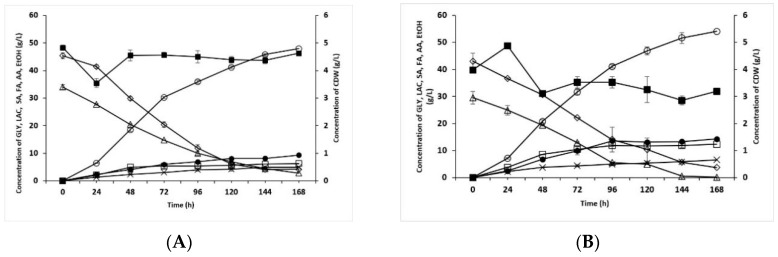
Time course of succinic acid (SA) production by batch fermentation using *Enterobacter* sp. LU1. (**A**) With glycerol (99.6%) and lactose. (**B**) With crude glycerol (85.0%) and whey permeate (77.0% of lactose). GLY (glycerol) (◊), LAC (lactose) (Δ), FA (formic acid) (x), AA (acetic acid) (□), EtOH (ethanol) (●), Biomass (■). The fermentation was carried out at 34 °C in a 2 L bioreactor with pH control by (A) automatic addition of 5% NaOH (*w*/*v*) and 20% Na_2_CO_3_ (*w*/*v*) or (B) manual addition of sterile MgCO_3_ to avoid pH decrease below 7.0. For the first 6 h, stirring was performed at 500 rpm with airflow at 1.0 vvm and was then decreased to 250 rpm without aeration. The values are the means of two independent samples.

Bioreactor culture on a medium containing 50 g/L of glycerol analytical grade and 25 g/L of lactose produced more than 35 g/L of succinic acid for the first time. This amount was obtained after 312 h of cultivation at 34 °C. The results obtained in the culture with an elevated level of lactose (32.5 g/L) are presented in Figure 1A. The final concentration of succinic acid in the culture medium was observed to increase to 48 g/L with the final performance of 0.58 g/g. Furthermore, this result was achieved after 168 h of culturing, which was almost two times faster than the time taken to reach a similar concentration in previous studies. The final productivity reached 0.29 g/L/h. Under the conditions described, glycerol was consumed significantly more quickly than lactose, and at the end of the culturing, the concentration of glycerol in the culture medium (slightly over 4 g/L) was very similar to that of lactose (almost 3 g/L). As before, the concentration of by-products (ethanol, formic acid, acetic acid) in the post-culture fluid did not exceed 21 g/L, with the highest concentration being noted for ethanol (ca. 9 g/L). It is likely that the aeration applied at the early stage of the *Enterobacter* sp. LU1 culturing enabled the growth of a large amount of bacterial biomass in excess of 4.5 g/L dry bacterial weight. The greatest increase in the concentration of bacterial biomass was observed at the early stage of culturing, which was likely due to the oxygen provided from the air during the 6 h aeration. Interestingly, even when biomass growth ceased and slightly decreased after 48 h, no decrease in succinic acid concentration was observed (its highest production rate was observed in the culture between 24 and 72 h). Another experiment was performed using crude glycerol and lactose originating from whey permeate. This culture achieved the highest concentration of succinic acid produced with the use of *Enterobacter* sp. LU1 (i.e., 54 g/L with the final performance and final productivity of 0.66 g/g and 0.32 g/L/h, respectively). Regarding substrate consumption, no significant differences were observed in the rate of glycerol consumption compared to culture with glycerol analytical grade, however, lactose was almost completely consumed (its concentration was below 1 g/L after 168 h of culturing). The concentration of the generated by-products was also higher than before, reaching almost 33 g/L. Although ethanol was again produced at the highest concentration (over 14 g/L), the concentration of acetic acid was not much less (over 12 g/L). The highest rate of bacterial biomass growth was observed during the aeration stage. Afterward, the growth was observed to decrease, which resulted in a slight decrease in biomass concentration over a 24 h period, which at the end of the culturing reached ca. 3.19 g/L dry weight. The highest concentration of biomass (ca. 4.87 g/L) was measured after 24 h of culturing (Figure 1B). 

### 3.2. Reduced YE Content and Different Methods of Inoculum Preparation

Although promising results were achieved in previous bioreactor cultures, their main drawback, from an economic perspective, was the use of YE as a source of nitrogen: added at a concentration of 15 g/L, YE significantly increases the cost of the succinic acid production process with the fermentation medium used in this study. The simultaneous statistical optimization of the fermentation medium confirmed the feasibility of succinic acid production by *Enterobacter* sp. LU1 on the culture medium without YE and with urea used as a source of nitrogen [26].

**Figure 2:**
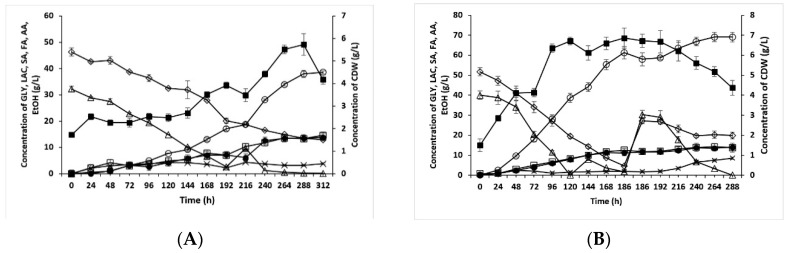
Time course of the fed-batch fermentation of SA on media containing decreased levels of yeast extract (YE). (**A**) With an initial aeration period during fermentation (first 4 h stirring at 500 rpm and airflow at 1.25 vvm, then stirring at 250 rpm and no aeration). (**B**) Without initial aeration at the beginning of fermentation (stirring at 250 rpm for the whole fermentation period). Both cultures were run using crude glycerol (85.0%) and whey permeate (77.0% of lactose). The fermentation was carried out at 34 °C in a 2 L bioreactor and pH was controlled by the manual addition of sterile MgCO_3_ to avoid a pH decrease below 7.0. The values are the means of two independent samples. GLY (glycerol) (◊), LAC (lactose) (Δ), FA (formic acid) (x), AA (acetic acid) (□), EtOH (ethanol) (●), Biomass (■).

Due to the culture conditions used (injection bottles, uncontrolled decrease of the pH of the fermentation fluid without supplementing MgCO_3_, culture time), we failed to obtain succinic acid concentrations above 20 g/L in the analyzed system. For this reason, further cultures were performed with the aim of producing succinic acid with a reduced amount of YE added to the medium under conditions of bioreactor cultures in order to achieve succinic acid production at a level similar to the best results obtained so far (i.e., around 50 g/L). Of all the conducted experiments, we here present the two that yielded the highest succinic acid production. Figure 2A shows the results for the first culture, which was terminated after almost 13 days. This fermentation produced over 38 g/L of succinic acid in 312 h. The highest volumetric rate of succinic acid production was observed on the tenth and eleventh days of culturing, and the maximum value reached almost 0.42 g/L/h. At the end of the culturing, the concentration of the by-products exceeded 30 g/L. The concentration of formate that was produced during the culturing initially increased in the culture medium until the 120^th^ hour before decreasing afterward until the 192nd hour. Changes in formate production occurred twice more during the culturing period. This was likely due to the periodic degradation of the produced formate to carbon dioxide and hydrogen. The rate of consumption of both substrates (i.e., glycerol and lactose) was similar throughout the culturing period, however, lactose was almost completely consumed whereas only ca. 1% of glycerol was consumed. The growth of bacterial biomass was observed to successively increase until the twelfth day and then decreased to ca. 4 g/L dry weight. 

The results obtained from the next culture are depicted in Figure 2B. After the 288th hour, this culture produced the highest concentration of succinic acid in the culture of *Enterobacter* sp. LU1 in this study, namely ca. 69 g/L. It is noteworthy that this result was obtained with a culture medium containing only 2 g/L of YE with urea being used as the main source of nitrogen at a dose of 4 g/L (fed in 1 g/L pulses throughout the culture period). Therefore, it can be concluded that this experiment confirmed the feasibility of the effective production of succinic acid by *Enterobacter* sp. LU1 on a culture medium with a reduced content of YE. This represents an extremely important step toward the potential industrial implementation of the described process. 

### 3.3. Microaerobic Cultivation on Crude Glycerol and Whey Permeate

**Figure 3:**
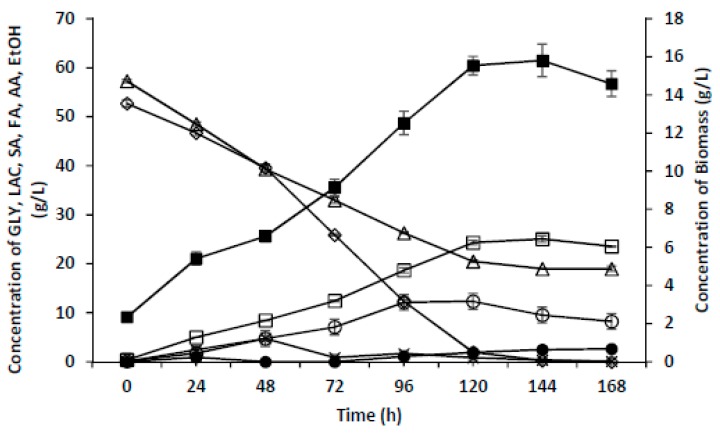
Time course of the microaerobic fermentation of SA. The fermentation was carried out using crude glycerol (85.0%) and whey permeate at 34 °C in a 2 L bioreactor with pH controlled by the manual addition of sterile MgCO_3_ to avoid a pH decrease below 7.0. The culture was stirred at 300 rpm and airflow was 0.25 vvm. The values are the means of two independent samples. GLY (glycerol) (◊), LAC (lactose) (Δ), FA (formic acid) (x), AA (acetic acid) (□), EtOH (ethanol) (●), Biomass (■).

In the present research, it was observed that, despite the very limited consumption of glycerol under anaerobic conditions, *Enterobacter* sp. LU1 was capable of effectively utilizing this source of carbon when oxygen was delivered to the culture system. Under such conditions, apart from an intensive growth of bacterial biomass, the main product of the process is usually acetic acid, although succinic acid may also be produced. Previous experiments carried out in bioreactors with intensive stirring (up to 1000 rpm) coupled with aeration failed to deliver the expected effective production of succinic acid caused by an increase in the number of bacteria in the system that served the role of biocatalysts in the production process. Despite the intensive consumption of glycerol as the only source of carbon, and despite the production of significant quantities of biomass, the bacteria cultured under conditions described in the paragraph above were incapable of producing succinic acid under anaerobic conditions. Considering the above, in the present study, an attempt was made to conduct experiments to investigate the possibility of succinic acid production by *Enterobacter* sp. LU1 under microaerophilic conditions. These experiments were carried out using bioreactor cultures of the discussed bacterial strain. As in previous cultures that were run under anaerobic conditions, glycerol and lactose were used as sources of carbon in the fermentation medium. Glycerol was added in the form of crude glycerol, while lactose was contained in whey permeate. After five days of *Enterobacter* sp. LU1 cultivation, the pH had begun to increase, only part of the lactose had been consumed, no increase was observed in the amount of products formed (succinic acid, acetic acid, formic acid, ethanol), and the bacteria had begun to utilize the produced organic acids. The detailed results achieved in this experiment are presented in Figure 3. These results indicate that under microaerophilic culture conditions, the LU1 strain consumes glycerol significantly faster than it consumes lactose. When the glycerol was completely consumed, the culture was terminated despite the presence of lactose in the medium (19 g/L). This was also the stage when the accumulated metabolites other than ethanol (succinic acid, formate, and acetate) began to be consumed. The results of this experiment revealed a high level of produced bacterial biomass (15.8 g/L) compared to the anaerobic cultures, with the amount of bacterial biomass decreasing slightly after the 144th hour.

### 3.4. Microaerobic Cultivation on Glycerol as the Sole Carbon Source

In another experiment run under semi-aerobic conditions, crude glycerol was used as the only source of carbon (i.e., without lactose). On the eighth day of the culture, the presence of succinic acid in the culture medium was noted at a level of 37 g/L with the final performance and final productivity of 0.74 g/g and 0.19 g/L/h, respectively. The detailed results of this culture are summarized in Table 1. This experiment confirmed the feasibility of producing succinic acid with a culture medium in which glycerol was used as the sole source of carbon (i.e., without the addition of lactose) under semi-aerobic conditions. Under such conditions, succinic acid was the largest product (37 g/L) of the completed culture, however, acetic acid production was not much lower (28 g/L). The rate of acetic acid production was highest at the beginning of the culturing, and acetic acid was the only metabolite formed until the 96th hour. This may be related to some extent to the inhibition of biomass growth, which decreased the production of succinic acid. Bacterial biomass increased until the 144th hour, after which a small decrease was observed in its growth. The final volume of produced biomass was negligibly lower than that of the previous culture, however, it was almost two times higher than in the anaerobic cultures. No ethanol production was observed throughout the culturing period. Interestingly, at the final stage of culturing, the amount of formic acid produced dropped to ca. 3 g/L. The maximum concentration of formic acid observed during the culturing was ca. 8 g/L. The results obtained after culture in medium containing glycerol analytical grade were lower than those obtained in medium with crude glycerol. On average, the concentration of succinic acid, process efficiency, and productivity were 30 g/L, 0.6 g/g, and 0.12 g/L/h, respectively. This experiment confirmed the feasibility of the effective production of succinic acid with a culture medium with glycerol without the necessity of adding lactose, albeit with the prerequisite of microaerophilic conditions.

## 4. Discussion

Although the capability of bacteria of the genus *Enterobacter* to produce large quantities of succinic acid has recently been reported in the literature [10], the quantities that were produced are relatively low compared to those obtained using most well-described bacterial producers of this acid [27]. One of the factors that allows higher concentrations of succinic acid to be produced in the post-culture liquid is the bioreactor culture conditions [28,29,30]. In the present study, succinic acid production to a level of 35 g/L was achieved in the first bioreactor culture run, in which glycerol analytical grade and lactose were added to the medium at doses of 50 g/L and 25 g/L, respectively. In a previous study, succinic acid production reached 0.51 g/g at a volumetric rate of acid production of ca. 0.11 g/L/h [10]. In the present study, an increase in succinic acid production by *Enterobacter* sp. LU1 was obtained in the bioreactor culture in which the lactose content in the medium was increased to 32.5 g/L and 6 h aeration of the medium was applied at the onset of the culturing; these modifications enabled a succinic acid production of 48 g/L almost twice as quickly as this level was obtained in previous studies using the same bacterial producer. Consequently, other culture parameters were also improved, in other words, the volumetric rate of acid production, which in this case reached almost 0.29 g/L/h, and succinic acid yield, which reached 0.58 g/g. The results obtained for this culture allow for the conclusion that the ability of *Enterobacter* sp. LU1 to produce succinic acid is not directly related to the growth stage of the bacterial culture, and that the production of succinic acid is possible during both bacterial cell division and during the stationary phase of culturing when cell division ceases. 

A further increase in the final concentration of succinic acid in the post-fermentation liquid was achieved by using crude glycerin instead of pure glycerol and whey permeate instead of pure lactose in the culture medium. These modifications, together with the neutralization of the produced acids with MgCO_3_ instead of the NaOH and Na_2_CO_3_ solution, allowed the production of over 54 g/L of succinic acid after 168 h of bioreactor culturing. The positive effect of replacing Na^+^ ions with Mg^2+^ ions as neutralizing agents has also been confirmed by other works describing the microbiological production of succinic acid [31]. Additionally, a previous study achieved improvements in the volumetric rate of succinic acid production and succinic acid yield, with these parameters reaching 0.32 g/L/h and over 0.66 g/g, respectively. In all three experiments described in the present paper, the maximum concentration of bacterial biomass was less than 4.9 g/L. The increase in bacterial biomass should contribute to an increase in the kinetic parameters of the succinic acid production process. The above-discussed cultures were run with the addition of 15 g/L of yeast extract, which makes the succinic acid production process highly cost-ineffective when conducted at a larger scale. In some previous studies, the application of yeast extract or peptone was found to be a key factor that maintained the ability for succinic acid production, especially with the use of glycerol as a source of carbon [9]. In order to reduce the cost of the succinic acid production process, other researchers have attempted to significantly decrease the addition of yeast extract in the culture medium. A study involving the optimization of the fermentation medium [26] demonstrated the feasibility of using *Enterobacter* sp. LU1 to produce succinic acid without the addition of a yeast extract. However, it failed to ensure the efficient production of this acid due to the applied culture conditions. Therefore, it is advisable to conduct bioreactor trials that eliminate the difficulties associated with running periodic cultures in bottles or glass flasks. In the present study, the addition of yeast extract in pulses to a total amount of 3 g/L as well as the addition of urea (3.5 g/L in total) and K_2_HPO_4_ (2.63 g/L in total) allowed for the production of over 38 g/L of succinic acid. An additional batch of whey permeate was added during the culture to compensate for lactose consumption. Despite the five-fold reduction in the amount of yeast extract added to the culture medium, the production of succinic acid still continued after 100 h of culturing. Decreasing the concentration of yeast extract slowed down succinic acid production (Q_SA_ = 0.12 g/L/h) and decreased the succinic acid yield to 0.44 g/L. This observation is consistent with the results of a previous study, which compared cultures run on minimal media and on media to which organic extracts had been added [27]. In another culture, the addition of yeast extract was reduced to 2 g/L and the step involving six hours of aeration at the preliminary stage of acid production was omitted. Urea, dipotassium hydrogen phosphate, and magnesium carbonate were all added during the culturing. Additionally, the culture medium was supplemented twice with whey permeate and once with a new batch of crude glycerol. The applied fermentation strategy allowed the highest concentration of succinic acid ever produced by *Enterobacter* sp. LU1 to be obtained. After 288 h of culturing, chromatographic analysis demonstrated the presence of 69 g/L of succinic acid in the culture medium. This concentration is comparable to that described in a patent application concerning the succinic acid production capability of a genetically modified strain of the bacterium *Basfia succiniciproducens* [24]. However, the strategy from BASF’s patent [24] applied in this study for *Enterobacter* sp. LU1 resulted in a lower volumetric rate of succinic acid production (0.24 g/L/h) and a lower succinic acid yield (0.58 g/g) than in the case of the German company. However, in [24], the biomass was produced separately and its concentration in the production medium was several times higher than in our study. In both our study and in [24], the maximum concentration of succinic acid in the post-fermentation liquid was 69 g/L. However, in the study that used bacteria of the family *Pasteurellaceae*, this concentration was obtained after genetic modification of the wild strain (*Basfia succiniciproducens* DD1). When bacteria of the genus *Basfia* were used for the production of succinic acid, considerably better kinetic parameters were obtained (Q_SA_ = 2.87 g/L/h; Y_SA_ = 1.15 g/g). However, this required the addition of yeast extract and/or peptone (as organic sources of nitrogen and vitamins) to a total concentration of 10 g/L, which is five times greater than the amount of yeast extract and/or peptone added in in present study using *Enterobacter* sp. LU1. Additionally, the production of succinic acid by bacteria of the species *Basfia succiniciproducens* requires the use of maltose as a co-substrate, which is significantly more expensive than the yeast extract. Nonetheless, the culture of *Enterobacter* sp. LU1 described in the present study confirmed the feasibility of producing considerable amounts of succinic acid on minimal media with the addition of a small amount of yeast extract (2 g/L). Such a capability has also been observed in *E. coli* and *Saccharomyces cerevisiae* [12,32]. Considering previous experiments involving anaerobic cultures of *Enterobacter* sp. LU1 and the intensive proliferation of this bacteria under aerobic conditions, in the present study, we investigated the possibility of producing succinic acid under semi-aerobic conditions. Our experiments were based on tests made on the culture medium that were similar to the tests that were applied earlier in a previous study under anaerobic conditions (i.e., with the addition of lactose in the form of whey permeate). However, under the test conditions in the present study, glycerol was preferred by the test bacteria (*Enterobacter* sp. LU1), and the main product of the process was acetic acid. Once glycerol had been completely consumed, signs of culture arrest were observed, despite the presence of lactose. Later in the culturing, the produced organic acids began to be consumed, first succinic acid and then acetic acid, however, lactose was not consumed. The observed decrease in the concentration of formic acid did not result from its assimilation, however, it could be due to its enzymatic degradation to hydrogen and carbon dioxide as a result of the effect of formate hydrogenlyase enzyme (FHL) [25]. In another culture in this study based on the same procedure, but under semi-aerobic conditions, glycerol alone was applied to the production medium. This modification resulted in an increased production of succinic acid (ca. 37 g/L), however, the acetic acid concentration reached ca. 28 g/L. The present study confirmed the feasibility of succinic acid production by *Enterobacter* sp. LU1 on glycerol alone without the necessity of supplementing the culture medium with lactose. Therefore, supplementation with lactose may not be required when fermentation is carried out under microaerobic conditions instead of anaerobic conditions. The improvement in glycerol consumption under microaerobic conditions observed in the present study is consistent with the results reported by Durnin et al. [33] for succinic acid production using *E. coli*. It is also noteworthy that, in the present study, the volume of bacterial biomass needed for succinic acid production was relatively low (5–7 g/L) compared to other producers of succinic acid such as *Corynebacterium glutamicum*, with which the effective volume of bacterial biomass ranges from 40 to 60 g/L dry weight [23].

In the present study, the newly isolated bacterial strain *Enterobacter* sp. LU1 was found to be an effective producer of succinic acid on culture media containing industrial substrates (i.e., crude glycerol and whey permeate), and a small amount of yeast extract. The results obtained in this study suggest that the use of *Enterobacter* sp. LU1 for the natural production of succinic acid on glycerol based media (Table 2) could become widespread around the world. However, to allow the full industrial competitiveness of such production, further research needs to be conducted to help accelerate the process described in this study through in-depth metabolomic analyses and systems biotechnological approaches.

## Figures and Tables

**Table 1:** Microaerobic succinic acid (SA) fermentation by *Enterobacter* sp. LU1 using crude glycerol as the sole carbon source.

Time (h)	GLY (g/L)	Products (g/L)				
		SA	FA	AA	EtOH	CDW
0	51.1 ± 0.49	0.26 ± 0.01	0.10 ± 0.02	0.30 ± 0.24	n.d.	1.45 ± 0.21
24	37.95 ± 0.62	2.50 ± 0.01	2.18 ± 0.05	5.20 ± 0.01	n.d.	4.88 ± 0.32
48	25.5 ± 0.47	6.77 ± 0.18	3.30 ± 0.14	9.10 ± 0.35	n.d.	6.90 ± 0.15
72	67.5 ± 0.03	11.51 ± 0.05	4.78 ± 0.05	12.96 ± 0.11	n.d.	7.94 ± 0.38
96	57.71 ± 1.28	15.69 ± 0.27	6.67 ± 0.14	16.68 ± 0.26	n.d.	8.89 ± 0.44
120	44.76 ± 0.63	21.73 ± 0.34	7.45 ± 0.02	19.95 ± 0.28	n.d.	8.31 ± 0.26
144	33.5 ± 0.19	28.19 ± 0.18	8.10 ± 0.03	23.00 ± 0.18	n.d.	11.18 ± 0.13
168	25.4 ± 0.29	33.12 ± 0.39	7.80 ± 0.16	25.60 ± 0.30	n.d.	11.40 ± 0.54
192	21.6 ± 0.48	37.37 ± 0.73	3.00 ± 0.06	28.80 ± 0.49	n.d.	10.23 ± 0.39

GLY—glycerol; SA—succinic acid; FA—formic acid; AA—acetic acid; EtOH—ethanol; CDW—cell dry weight.

**Table 2:** Comparison of succinic acid (SA) production on glycerol using different microorganisms.

Strain	Fermentation Conditions	Concentration (g/L)	Yield (g/g)	Productivity (g/L/h)	Reference
*Actinobacillus succinogenes* NCIMB 41825	Anaerobic, batch	29.3	1.23	0.270	[9]
*Anaerobiospirillum succiniciproducens* ATCC 53488	Anaerobic, fed-batch (feeding with YE and glycerol)	19	1.60	0.157	[3]
*Escherichia coli pck*^+^ Δ*pfl*	Anaerobic, batch	12.1	1.03	0.084	[34]
*Yarrowia lipolytica* Y3314 Δ*sdh*2	Aerobic, batch	45	0.36	0.271	[35]
Basfia succiniciproducens DD1	Anaerobic, batch (without disaccharide addition)	19.5	1.12	0.81	[24]
*Basfia succiniciproducens* DD1 Δ*pfl* Δ*ldh*	Anaerobic, batch (without disaccharide addition)	36.2	1.26	1.50	[24]
*Basfia succiniciproducens* DD1 Δ*pfl* Δ*ldh*	Anaerobic, batch (maltose addition)	69.8	1.11	2.90	[24]
*Enterobacter* sp. LU1	Microaerobic, fed-batch	37.3 ± 0.73	0.38 ± 0.21	0.19 ± 0.09	This study
*Enterobacter* sp. LU1	Anaerobic, fed-batch (lactose addition)	69.0 ± 2.50	0.58 ± 0.11	0.24 ± 0.12	This study
